# Editorial: Wound Recognition across the Tree of Life

**DOI:** 10.3389/fpls.2016.01319

**Published:** 2016-09-01

**Authors:** Martin Heil, Walter G. Land, Mahmut Tör

**Affiliations:** ^1^Departamento de Ingeniería Genética, Centro de Investigación y de Estudios Avanzados del Instituto Politécnico Nacional - Unidad IrapuatoIrapuato, Mexico; ^2^Laboratoire d'Immuno Rhumatologie Moléculaire, INSERM UMR_S1109, Faculté de Médecine, Université de StrasbourgStrasbourg, France; ^3^Institute of Science and the Environment, University of WorcesterWorcester, UK

**Keywords:** damaged-self recognition, DAMPs (damage-associated molecular patterns), innate immunity, adaptive immunity, priming

All multi-cellular organisms share the necessity to perceive damage and to employ an adequate immune response to withstand injury and infection. The role of damage-associated molecular patterns (DAMPs) in the mammalian adaptive immune system and in allograft rejection was discovered by Polly Matzinger and Walter Land (Land et al., 1994; Matzinger, 1994). These discoveries revolutionized the research into transplantation and immunity (Land et al., 2016a,b) and improved the understanding of chronic and inflammation-related diseases such as Alzheimer's disease, Diabetes, Lupus, Rheuma (Land, 2015a,b), and many forms of cancer (Land, 2015c; Candeias and Gaipl, 2016). Unfortunately, the tendency toward specialization in contemporary science, albeit allowing for an incredible increase in the efficiency at which knowledge is being generated, enhances the risk to lose the communication across disciplines. A prime example of this situation is the research into injury perception and immunity, which developed in distinct disciplines for mammals and plants. In consequence, the first application of the DAMPs concept to plants appeared 13 years after their first description for mammals (Lotze et al., 2007). Two years later, four review papers discussed the role of DAMPs and “damaged-self recognition” in plants (Boller and Felix, 2009; Heil, 2009; Metraux et al., 2009; Tör et al., 2009).

In an attempt to close this gap, ‘DAMPs, 2016’ the first international and trans-disciplinary congress on injury perception and immunity, aims at promoting the trans-disciplinary research into wound recognition in organisms across the tree of life. A central step toward a better cross-disciplinary communication in this field was the Research Topic “Wound recognition across the tree of life.” Eleven articles co-authored by 43 researchers were published between July and November 2014 and attracted over 55,000 views by now (http://journal.frontiersin.org/researchtopic/2173/wound-recognition-across-the-tree-of-life). Reviews summarized the functions of DAMPs in insects (Krautz et al.) and plants (Savatin et al.), applied the “danger model” to mosquitoes (Moreno-García et al.), and discussed the role of extracellular ATP (eATP) as a DAMP in plants (Tanaka et al.). It was known before that eATP induces plant defense (Roux and Steinebrunner, 2007; Chivasa et al., 2009; Heil et al., 2012), but only the discovery of its specific receptor (Choi et al., 2014) provided unambiguous support for a role of eATP as a DAMP (Tanaka et al.). Interestingly, eATP also acts as DAMP in the fungus, *Trichoderma viride* (Medina-Castellanos et al.).

Three papers reported how Arabidopsis responds to enemies with different degrees of specialization and combined transcriptional with metabolomic data to distinguish responses to a chewing insect vs. bacterial infection (Appel et al.; Appel et al.; Rehrig et al.). Responses to an aphid and a caterpillar shared only a surprising 10% of the up-regulated and 8% of the down-regulated genes, and even responses to caterpillars from different species (*Spodoptera exigua* and *Pieris rapae*) shared only 21% of the up-regulated and 12% of the down-regulated genes (Appel et al.). Transcriptional changes were frequently weaker or absent in response to the specialist (*P. rapae*; Rehrig et al.). The degree to which DAMPs contribute to this specificity remains subject of speculation (Duran-Flores and Heil, 2016), although responses of bean to leaf homogenates from various species demonstrated specificity when plants only perceive endogenous “danger signals” (Duran-Flores and Heil). A fine-tuning of plant defenses was also reported for methanol, a wound-generated molecule that functions in within- and between-plant signaling (Dorokhov et al., 2012; Komarova et al.) and strongly modulated the response of Arabidopsis and Tomato to pathogen-associated molecular patterns (PAMPs; Hann et al.).

Recent studies, mostly published after the Research Tropic, revealed multiple similarities between “trained immunity” in mammals (Crişan et al., 2016) and resistance “priming” in plants (Martinez-Medina et al., 2016). Firstly, parasites of mammals, plants, and insects frequently target the same processes to manipulate host immunity (Guiguet et al., 2016; Heil), and mammals, insects, plants, and fungi respond to damage employing similar mechanisms (Hernández-Oñate and Herrera-Estrella, 2015). Secondly, preparing the immune system for more efficient responses (“priming”) appears to be a general feature of DAMPs (Crişan et al., 2016; Martinez-Medina et al., 2016). The perception of DAMPs initiates the maturation of dendritic cells to antigen-presenting cells (Matzinger, 2002) and gene expression for the *NOD-Like Receptor family Protein* 3 (NLRP3)-inflammasome in macrophages. Consecutive sensing of DAMPs or PAMPs by NLRP3 activates the inflammasome (Figure 4 in Heil and Land). Thus, DAMPs prime the immune system for more directed and sensitive responses to future problems. At least in plants, this effect depends on epigenetic alterations and can last into the next generation (Rasmann et al., 2012). Thirdly, many DAMPs exert a double-function as direct anti-microbial compound and signal. For example, mammalian type-I Interferons have antiviral effects, and plant secondary compounds such as DIMBOA, various glucosinolate breakdown products and herbivore-induced plant volatiles are quickly synthesised—or released from stored precursors—when plants, are damaged and have both, biocidal and signaling (immunity enhancing) activity (Gallucci and Matzinger, 2001; Ahmad et al., 2011; Andersson et al., 2015; Veyrat et al., 2016).

Finally, the development of ROS and the involvement of NADPH oxidase, Ca^2+^ influxes and downstream MAPKinase signaling cascades are common features of DAMP-induced immune responses in organisms across the tree of life (Duran-Flores and Heil; Medina-Castellanos et al.; Crişan et al., 2016; Segal, 2016). An inhibitor of the mammalian NADPH oxidase inhibited ROS development in plants (Dwyer et al., 1995; Tenhaken et al., 1995), anti-sera to key mammalian proteins cross-reacted with the respective plant proteins (Dwyer et al., 1995; Tenhaken et al., 1995), and the human DAMP, high mobility group box (HMGB) protein 3, activated immunity in plants (Choi et al., 2016). Immunological and pharmacological cross-reactions make homology likely in these cases. By contrast, the function of eATP as a DAMP in plants, fungi, and mammals appears to be the result of independent evolution, because eATP receptors in mammals and plants belong to different families (Choi et al., 2014). In summary, we conclude that damaged-self recognition and the involved perception mechanisms and signaling pathways contain both homologous and analogous elements among plants and mammals (Heil and Land).

## Author contributions

MH prepared a first draft of the manuscript and all authors listed have made substantial, direct and intellectual contribution to the work, and approved it for publication.

### Conflict of interest statement

The authors declare that the research was conducted in the absence of any commercial or financial relationships that could be construed as a potential conflict of interest.
